# Compliance of patients to DOTS tuberculosis treatment strategy in a South-East Nigeria Teaching Hospital

**DOI:** 10.4314/ahs.v22i3.64

**Published:** 2022-09

**Authors:** Chinedu A Idoko, Olufemi Adeyemi

**Affiliations:** 1 Department of Community Medicine, University of Nigeria, Enugu; 2 UNICEF Field Office, Enugu, Nigeria

**Keywords:** Compliance, Patients, DOTS, Tuberculosis, South-East Nigeria

## Abstract

**Introduction:**

Tuberculosis ranks the second highest cause of adult mortality after HIV in the world. The Directly Observed Treatment Short course (DOTS) strategy is aimed at following up on patients' adherence to treatment regimen.

**Objectives:**

To assess the level of compliance of patients to the DOTS strategy.

**Materials and Methods:**

A retrospective study of patients seen at the University of Nigeria Teaching Hospital from January, 2013 to April, 2015. Relevant information was collected from patients' folders. Data analysis was with the SPSS and results represented in tables.

**Results:**

111 (50%) patients were compliant with their DOTS treatment plan while 107 (41.3%) were non-compliant. Ninety-two patients (41.4%) were successfully treated and discharged home, 7 patients (3.2%) referred to other centres. The proportion of patients regarding their marital status, occupation, educational level and address that was compliant to the DOTS TB reflected varied patterns.

**Conclusion/ Recommendations:**

The study reflected poor to average compliance to DOTS. There is a need for creation of more DOTS centres; regular surveys and updates on DOTS TB strategy should be the norm rather than the exception.

## Introduction

Tuberculosis is an infectious airborne disease and a major global health concern. It is caused by the organism Mycobacterium tuberculosis. It affects all countries but 85% of cases occur in Africa and Asia.^1^

Globally there are 22 High Burden Countries (HBC) that account for 80% of the world's TB cases. These countries include Afghanistan, Bangladesh, Brazil, Cambodia, China, Congo, Ethiopia, India, Indonesia, Kenya, Mozambique, Myanmar, Nigeria, Pakistan, Philippines, Russia, South Africa, Thailand, Uganda, Tanzania, Vietnam, and Zimbabwe. Nigeria ranks tenth among this 22 HBC around the world and 4^th^ in Africa^1^.

According to the WHO estimates in 2011, Nigeria has an estimated 320,000 cases of all forms of TB with a yearly prevalence of 199 cases per 100,000 population. TB ranks the second highest cause of adult mortality after HIV in the world^2^. It has male sex preponderance, most common among the economically productive age group^1^. Tuberculosis has attracted strong political interest over the years and this has led to the establishment of the National Tuberculosis and Leprosy Control Program (NTBLCP), an arm of the Federal Ministry of Health that has been given the mandate to control TB and leprosy in Nigeria. The vision of the program is “Nigeria free of TB”, while the goal is to reduce TB to a level whereby it is no longer a disease of public health importance^3^, ^4^. In line with this vision, the WHO adopted the Directly Observed Treatment Short course (DOTS) strategy (aimed at following up on patients' adherence to treatment regimen) in 1990, while Nigeria adopted it in 1993. It has so far been implemented in all the 36 states including FCT^5^,^6^.

This study was therefore conducted to assess the level of compliance of patients to DOTS in the chest clinic (old site) of the University of Nigeria Teaching Hospital (UNTH) from 2013-2015.

There has been a 47% drop in the death rate of tuberculosis patients since the introduction of the DOTS strategy 5 showing that the strategy is effective globally. Nigeria, however, remained among the top 6 countriesof the 22 high burden countries in the world^5^. While Nigeria and other countries are making progress; this study was done to find out why Nigeria remains among the top 6, despite applying same strategy.

## Study objectives

The general objective of the study was to assess the level of compliance of patients to DOTS in Nigeria using the University of Nigeria Teaching Hospital chest clinic. The specific of objectives included: to determine the treatment outcome of patients on DOTS TB; and to establish the reasons for noncompliance of some patients to the DOTS strategy.

## Materials and methods

### Study area

The study was conducted at the chest clinic of University of Nigeria Teaching Hospital, Enugu. University of Nigeria Teaching Hospital has different outlets; the permanent site at Ituku Ozalla in Nkanu East/West in Enugu State, the Old site at old UNTH road Enugu North LGA and the Comprehensive Health centre sited at Obukpa in Nsukka LGA.

Enugu is the State capital with a population of 3.8 million people. The Chest Clinic is run by the Community Medicine Department UNTH and runs every Monday, Wednesday, and Friday with an average attendance of 15–20 patients per week.

### Study design

The study was a retrospective study of patients seen at the UNTH chest clinic from January, 2013 to April, 2015. Patients' folders were retrieved from the medical records department of the chest clinic and relevant information collected for the study.

### Study population

The study population was the tuberculosis patients (male and female of all ages) who attended the chest clinic of UNTH within the time period.

### Sample size

Based on a previous and related study, the sample size was derived using the formula

N=Z^2^P(1-P)/D^2^

Where N is the normal/sample size for the study,

P=64 % (compliance rate in Nepal by Shiyalap 2013) 7

Z=1.96, with a 95% confidence limit

D (sampling error) = 5 % (0.05) which is the tolerance error or margin of error tolerated.

Hence, N=1.96x0.64 (1–0.64)/0.052

1.2544x0.36/0.0025

=180.4

=180

Taking into account 10% attrition, this gave a minimum sample size of 198 which was approximated to 200.

Sample size however used in this study is 222 patients.

### Statistical analysis

Statistical analysis was done using the Statistical Package for the Social Sciences (SPSS) and results displayed in tables.

## Results

Table 1 show that a total of 222 patients received tuberculosis treatment using DOTS strategy at the chest clinic of University of Nigeria Teaching Hospital, Enugu from 2013 to 2015. One hundred and nineteen (53.6%) of them were males. Their ages ranged from 2 to 85 years with a mean age of 38 years. The 21–30 years age group was the most commonly represented with 71 (32%) patients and the >80 years group was the least represented with 2 (0.9%) patients.

**Table 1:** Social demographic characteristics of studied populations

Age	Frequency	Sex		Marital status			Formal Education	
		Male	Female	Single	Married	Widowed	Yes	No
1–10	6	2	4	6			2	
11–20	23	9	14	22	1		9	
21–30	71	28	43	52	18	1	28	
31–40	49	34	15	23	26		35	
41–50	36	24	12	3	33		25	
51–60	21	13	8	2	18	1	13	
61–70	11	7	4	2	9		6	1
71–80	3	1	2	1	2			1
>80	2	1	1	1		1		1
Total	222	119	103	112	107	3	118	3

Table 2 shows that during the course of DOTS treatment, 111 (50%) patients were compliant with their DOTS treatment plan while 107 (41.3%) were non-compliant defaulting at different times during the course of treatment. Four (1.8%) patients were referred and their compliance could not be determined while 17(7.7%) patients died during the course of treatment.

**Table 2:** Compliance to treatment of patients using dots strategy for TB treatment

	Compliance to DOTS		Frequency	Total	Percentage	Percentage
Compliant			111	111	50	50
**Non-Compliant**	**Alive**	Default at 1^st^ visit	18	107	8.1	41.3
		Default at 1mth	15		6.8	
		Default at 2mths	21		9.5	
		Default at 3mths	9		4.1	
		Default at 4mths	13		5.9	
		Default at 5mths	15		6.9	
	**Dead**	Died after 1mth	8		3.6	7.7
		Died after 2mth	4		1.8	
		Died after 3mth	2		0.9	
		Died after 4mth	1		0.5	
		Died after 5mth	1		0.5	
**Referred**			4	4	1.4	1.0
**Total**			222	222	100	100

In Table 3, it can be seen that 92 (41.4%) patients were successfully treated and discharged home, 7 (3.2%) patients were referred to other centres. Eleven 1(4.9%) patients completed treatment course but did not show up for discharge, and 3(1.4%) had a relapse. While 89 (40.1%) patients had an unknown outcome, 17(7.7%) patients died and 3 (1.4%) were still on treatment.

**Table 3:** Outcome of treatment of patients using DOTS TB

Outcome of treatment	Frequency	Percentage
Referred	7	3.2
Discharged	92	41.2
Didn't show up for discharge	11	4.9
Still on treatment	3	1.4
Dead	17	7.7
Unknown	89	40.1
Relapse	3	1.4
Total	222	100.0

Table 4: Here, the proportion of the single, married and widowed compliant to treatment using DOTS TB showed the highest compliance among the single with a frequency of 65 (58.5%) patients, while 55.6% of the married were less compliant.

**Table 4:** Proportion of the single, married and widowed patients compliant to treatment using dots TB

Status	Compliant	Percentage	Noncompliant	Percentage
Single	65	58.5	43	43.6
Married	45	40.5	59	55.6
Widowed	1	0.9	2	1.9
Total	111	100	104	100

The sum total on this table = 215

This is because there were 7 cases (3 married and 4 singles) that were referred and so their compliance couldn't be determined

This makes it a total of 222

Table 5 is concerned with the occupational distribution. Civil servants 37 (33.3%), were most compliant followed by students 26 (23.4%), businessmen/traders 25 (22.5%), unemployed 19 (17.1%), and farmers 4 (3.6%). Noncompliance was highest amongst the unemployed 36 (33.9%), followed by students 29 (29.2%), businessmen/traders 26 (24.5%) civil servants 9 (8.4%), and farmers 4 (3.8%).

**Table 5:** Proportion of patients that are unemployed, students/ undergraduates; and of different occupations compliant to treatment using DOTS TB

Occupation	Compliant	Percentage	Noncompliant	Percentage
Unemployed	**19**	**17.1**	**36**	**33.9**
Student/undergraduate	**26**	**23.4**	**29**	**29.2**
Farmer	**4**	**3.6**	**4**	**3.8**
Civil servant	**37**	**33.3**	**9**	**8.4**
Business/trader	**25**	**22.5**	**26**	**24.5**
**Total**	**111**	**100**	**104**	**100**

The sum total on this table = 215

This is because there were 7 cases (2 farmers, 4 students, and 1 trader) that were referred and so their compliance couldn't be determined

This makes it a total of 222

Table 6 shows that those who had tertiary education proved the highest proportion of compliant patients to the DOTS strategy 56 (50.5%), followed by those with post primary education 30 (27%), primary school graduates: 20 patients (18%) and then patients with no formal education 5 (4.5%).

**Table 6:** Proportion of the non-educated patients and patients of different literacy levels compliant to treatment using DOTS TB

Educational status	Compliant	Percentage	Noncompliant	Percentage
None	**5**	**4.5**	**4**	**3.8**
Primary	**20**	**18**	**17**	**16.3**
Secondary	**30**	**27**	**74**	**71.2**
Tertiary	**56**	**50.5**	**9**	**8.7**
**Total**	**111**	**100**	**104**	**100**

The sum total on this table = 215

This is because there were 7 cases (2 none, 1 primary and 4 secondary) that were referred and so their compliance couldn't be determined

This makes it a total of 222

Table 7 captures the proportion of patients regarding their address. Compliance was 73.9% amongst those living within Enugu metropolis, followed by those living outside the city but within the state 26 (23.4%) and those living outside the state 3 (2.7%).

**Table 7:** Proportion of patients regards their location of abode that were compliant to treatment using DOTS

Location of abode	Compliant	Percentage	Noncompliant	Percentage
Enugu city	**82**	**73.9**	**65**	**62.5**
Outside Enugu city but within Enugu state	**26**	**23.4**	**32**	**30.8**
Outside Enugu state	**3**	**2.7**	**7**	**6.7**
**Total**	**111**	**100**	**104**	**100**

The sum total on this table = 215

This is because there were 7 cases (5 were from outside the state and 2 were from within the state) that were referred and so their compliance couldn't be determined

This makes it a total of 222

## Discussion

Tuberculosis control programs currently emphasize the DOTS Strategy, promoted by the World Health Organization (WHO) and the International Union against Tuberculosis and Lung Disease (IUATLD) 8,9,10.

Direct observation and supervision of patients is assumed to be more effective than self-administration to ensure that patients successfully complete the recommended 6–9 months Chemotherapy.

Our study involved the review of 222 folders of patients, out of which 119 (53.6%) were males. Ninety-two (41.2%) of the patients seen in the period of the study were successfully treated and discharged as cured. One hundred and seven (48.2%) patients were noncompliant at some point in the course of their treatment. Out of the 107 noncompliant patients, 17(7.7%) patients died, and these were HIV positive patients who were non-complaint with treatment. This is consistent with the findings of Amoran et al who noted that the HIV positive patients that were non-complaint with medications also died in his study in Ogun State, Nigeria 11.

The age range of the patients studied was 2–85yrs (mean age 38). Compliance to DOTS strategy was found to be highest at the age group of 21–60yrs (80.4%), which is the economically productive age group. The age group with the highest compliance is consistent with the findings of Pandel^12^ who noted the highest compliance of the age group 15–59 years to be 84%. In his study in Nepal, compliance was higher in this age group, possibly because these were active, strong and could always get to the clinic when they wanted to. Also, they were the economically and financially stable group, so transportation was not a problem for them.

A total of 177 (41.3%) of the 222 patients defaulted in their treatment. The default was highest at 2months that is: 21 patients (9.5%), which is almost consistent with the findings of Amoran et al who noted a default rate of 12.6% at the 2nd month in their study in Ogun State. This could be due to not being able to cope with the side effects of the drugs. A 41.3% default rate is not consistent with the findings of Inotu et al who showed a default rate of 23.8% in their study in Benin City, Nigeria^13^. This shows that compliance is low in this area and is due to certain factors which were also discovered in the course of the current study and included - distance from the clinic, educational status, occupation, awareness of the disease and its progression.

In our study, compliance was higher amongst the singles (58.5%) than against the married (40.5%). This is consistent with the findings of Boyle who found higher compliance amongst the singles^14^. This could be due to the added responsibility of providing and caring for children of the married couples which prove sources of distractions for the parent patients which may make them skip their drugs or clinic visits.

Regarding occupation of the patients, the highest rate of compliance was found amongst the civil servants 37 (28.8%) and noncompliance highest amongst the unemployed 36 (33.9%). These are similar to the findings of Erhabor et al who noted highest compliance rate among the unemployed and lowest among the civil servants^15^. The civil servants were the most compliant in our study, possibly because of their level of education and it was easier for them to obtain permission from work to go for their check-up appointments. Furthermore, their profession instils discipline into the lives of workers so they tend to adhere to their drug regimen while following up with check-ups. The unemployed had the lowest compliance which is attributable to several factors that could include lack of transport fare, depression/ frustration arising from their unemployed state.

Regarding educational level, it was discovered that compliance was highest amongst those that attained tertiary education (53.2%), while noncompliance was highest amongst those with secondary education (71.2). These findings are not consistent with findings of Pandit who discovered that compliance was highest among those who only attained primary education and lowest among graduates in his study in India^16^. High compliance among tertiary institution certificate holders could be hinged on the expected commensurate attained knowledge and exposure. This class of persons could easily surf the internet for information and knowledge regarding treatment, drug side effects and dangers of non-compliance. Furthermore, low compliance among post primary school certificate holders could be related to limited knowledge. Some of them are probably still students with attendant issues of youthful exuberance. For others, distraction of business and other job-related indulgence may be contributory.

On considerations of patients' location of abode and treatment compliance, it was found (and expectedly so) that the highest compliance was with those living within Enugu metropolis (73.9%) and lowest amongst those living outside the state (2.7%) which is consistent with the finding of Vieira et al who found that those living far from the clinics were less compliant^17^. This is could be as a result of the stress of long distance to treatment points. Furthermore, the cost of transportation and the low standard of living are strong associations for tuberculosis. Bad road networks linking rural and urban areas remain a limiting factor to clinics accessibility.

## Conclusion and Recommendation

A significant number of patients were not compliant to DOTS at the study clinic. Periodic surveys/ studies to assess compliance and proffer solutions to making DOTS more effective would be helpful. The issue of location of abode/ distance to clinic to compliance can be addressed by creating more DOTS centres in the rural areas. Regular surveys and updates on DOTS TB Strategy should be the norm rather than exception. There should be increased awareness on tuberculosis using both the formal and informal media. The importance of involving patients' family members in following up on their treatment and compliance cannot be overemphasized.
